# Liquid crystal display and organic light-emitting diode display: present status and future perspectives

**DOI:** 10.1038/lsa.2017.168

**Published:** 2018-03-23

**Authors:** Hai-Wei Chen, Jiun-Haw Lee, Bo-Yen Lin, Stanley Chen, Shin-Tson Wu

**Affiliations:** 1 College of Optics and Photonics, University of Central Florida, Orlando, FL 32816, USA; 2 Graduate Institute of Photonics and Optoelectronics and Department of Electrical Engineering, Taiwan University; 3 Nichem Fine Technology Co. Ltd.

**Keywords:** ambient contrast ratio, liquid crystal displays, motion picture response time, organic light-emitting diode

## Abstract

Recently, ‘Liquid crystal display (LCD) vs. organic light-emitting diode (OLED) display: who wins?’ has become a topic of heated debate. In this review, we perform a systematic and comparative study of these two flat panel display technologies. First, we review recent advances in LCDs and OLEDs, including material development, device configuration and system integration. Next we analyze and compare their performances by six key display metrics: response time, contrast ratio, color gamut, lifetime, power efficiency, and panel flexibility. In this section, we focus on two key parameters: motion picture response time (MPRT) and ambient contrast ratio (ACR), which dramatically affect image quality in practical application scenarios. MPRT determines the image blur of a moving picture, and ACR governs the perceived image contrast under ambient lighting conditions. It is intriguing that LCD can achieve comparable or even slightly better MPRT and ACR than OLED, although its response time and contrast ratio are generally perceived to be much inferior to those of OLED. Finally, three future trends are highlighted, including high dynamic range, virtual reality/augmented reality and smart displays with versatile functions.

## Introduction

Display technology has gradually but profoundly shaped the lifestyle of human beings, which is widely recognized as an indispensable part of the modern world^1^. Presently, liquid crystal displays (LCDs) are the dominant technology, with applications spanning smartphones, tablets, computer monitors, televisions (TVs), to data projectors^2, 3, 4, 5^. However, in recent years, the market for organic light-emitting diode (OLED) displays has grown rapidly and has started to challenge LCDs in all applications, especially in the small-sized display market^6, 7, 8^. Lately, ‘LCD vs. OLED: who wins?’ has become a topic of heated debate^9^.

LCDs are non-emissive, and their invention can be traced back to the 1960s and early 1970s^10, 11, 12, 13, 14, 15^. With extensive material research and development, device innovation and heavy investment on advanced manufacturing technologies, thin-film transistor (TFT) LCD technology has gradually matured in all aspects; some key hurdles, such as the viewing angle, response time and color gamut, have been overcome^5^. Compared with OLEDs, LCDs have advantages in lifetime, cost, resolution density and peak brightness^16^. On the other hand, OLEDs are emissive; their inherent advantages are obvious, such as true black state, fast response time and an ultra-thin profile, which enables flexible displays^8, 9^. As for color performance, OLEDs have a wider color gamut over LCDs employing a white light-emitting diode (WLED) as a backlight. Nevertheless, LCD with a quantum dot (QD) backlight has been developed and promoted^17, 18, 19, 20^. The full width at half maximum (FWHM) of green and red QDs is only 25 nm. As a result, a QD-enhanced LCD has a wider color gamut than an OLED. Generally speaking, both technologies have their own pros and cons. The competition is getting fierce; therefore, an objective systematic analysis and comparison on these two superb technologies is in great demand.

In this review paper, we present recent progress on LCDs and OLEDs regarding materials, device structures to final panel performances. First, in Section II, we briefly describe the device configurations and operation principles of these two technologies. Then, in Section III, we choose six key metrics: response time, contrast ratio, color gamut, lifetime, power efficiency, and panel flexibility, to evaluate LCDs and OLEDs. Their future perspectives are discussed in Section IV, including high dynamic range (HDR), virtual reality/augmented reality (VR/AR) and smart displays with versatile functions.

## Device configurations and operation principles

### Liquid crystal displays

**Figure 1:**
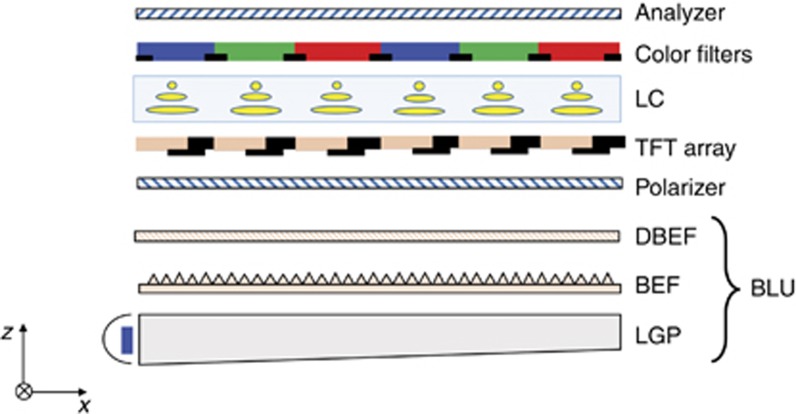
Schematic diagram of an LCD. BEF, brightness enhancement film; BLU, backlight unit; DBEF, dual brightness enhancement film; LGP, light guide plate.

Liquid crystal (LC) materials do not emit light; therefore, a backlight unit is usually needed (except in reflective displays) to illuminate the display panel. Figure 1 depicts an edge-lit TFT-LCD. The incident LED passes through the light-guide plate and multiple films and is then modulated by the LC layer sandwiched between two crossed polarizers^5^. In general, four popular LCD operation modes are used depending on the molecular alignments and electrode configurations: (1) twisted nematic (TN) mode, (2) vertical alignment (VA) mode, (3) in-plane switching (IPS) mode, and (4) fringe-field switching (FFS) mode^13, 14, 15, 21^. Below, we will briefly discuss each operation mode.

#### TN mode

**Figure 2:**
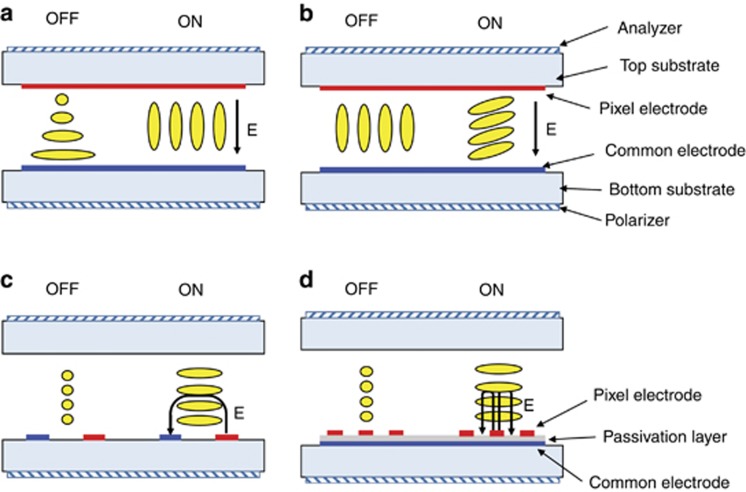
Schematic diagram of the (**a**) TN mode, (**b**) VA mode, (**c**) IPS mode and (**d**) FFS mode. The LC director orientations are shown in the voltage-off (left) and voltage-on (right) states.

The 90° TN mode was first published in 1971 by Schadt and Helfrich^13^. In the voltage-off state, the LC director twists 90° continually from the top to the bottom substrates (Figure 2a), introducing a so-called polarization rotation effect. As the voltage exceeds a threshold (*V*_th_), the LC directors start to unwind and the polarization rotation effect gradually diminishes, leading to decreased transmittance. This TN mode has a high transmittance and low operation voltage (~5 *V*_rms_), but its viewing angle is somewhat limited^22^. To improve the viewing angle and extend its applications to desktop computers and TVs, some specially designed compensation films, such as discotic film or Fuji film, are commonly used^23, 24^. Recently, Sharp developed a special micro-tube film to further widen the viewing angle and ambient contrast ratio (ACR) for TN LCDs^25^.

#### VA mode

VA was first invented in 1971 by Schiekel and Fahrenschon^14^ but did not receive widespread attention until the late 1990s, when multi-domain VA (MVA) mode was proposed to solve the viewing angle problem^26, 27, 28^. In the VA mode, an LC with a negative Δ*ε*<0 is used and the electric field is in the longitudinal direction. In the initial state (*V*=0), the LC directors are aligned in the vertical direction (Figure 2b). As the voltage exceeds a threshold, the LC directors are gradually tilted so that the incident light transmits through the crossed polarizers. Film-compensated MVA mode has a high on-axis contrast ratio (CR; >5000:1), wide viewing angle and fairly fast response time (5 ms). Thus it is widely used in large TVs^29, 30^. Recently, curved MVA LCD TVs have become popular because VA mode enables the smallest bending curvature compared with other LCDs^31, 32^.

#### IPS mode

IPS mode was first proposed in 1973 by Soref^15^ but remained a scientific curiosity until the mid-1990s owing to the demand of touch panels^33, 34^. In an IPS cell, the LC directors are homogeneously aligned and the electric fields are in the lateral direction (Figure 2c). As the voltage increases, the strong in-plane fringing electric fields between the interdigital electrodes reorient the LC directors. Such a unique mechanism makes IPS a favorable candidate for touch panels because no ripple effect occurs upon touching the panel. However, the peak transmittance of IPS is relatively low (~75%) because the LC molecules above the electrodes cannot be effectively reoriented. This low transmittance region is called a dead zone^5^.

#### FFS mode

FFS mode was proposed in 1998 by three Korean scientists: SH Lee, SL Lee, and HY Kim^21^. Soon after its invention, FFS became a popular LCD mode due to its outstanding features, including high transmittance, wide viewing angle, weak color shift, built-in storage capacitance, and robustness to touch pressure^35, 36, 37^. Basically, FFS shares a similar working principle with IPS, but the pixel and common electrodes are separated by a thin passivation layer (Figure 2d). As a result, the electrode width and gap are able to be much smaller than those of IPS, leading to much stronger fringe fields covering both the electrode and gap regions. Thus the dead zone areas are reduced. In general, both positive (p-FFS) and negative (n-FFS) Δ*ε* LCs can be used in the FFS mode^38, 39^. Currently, n-FFS is preferred for mobile applications because its transmittance is higher than that of p-FFS (98 vs. 88%)^40^.

As summarized in Table 1, these four LCD modes have their own unique features and are used for different applications. For example, TN has the advantages of low cost and high optical efficiency; thus, it is mostly used in wristwatches, signage and laptop computers, for which a wide view is not absolutely necessary. MVA mode is particularly attractive for large TVs because a fast response time, high CR and wide viewing angle are required to display motion pictures. On the other hand, IPS and FFS modes are used in mobile displays, where low power consumption for a long battery life and pressure resistance for touch screens are critical.

### Organic light-emitting diode

**Figure 3:**
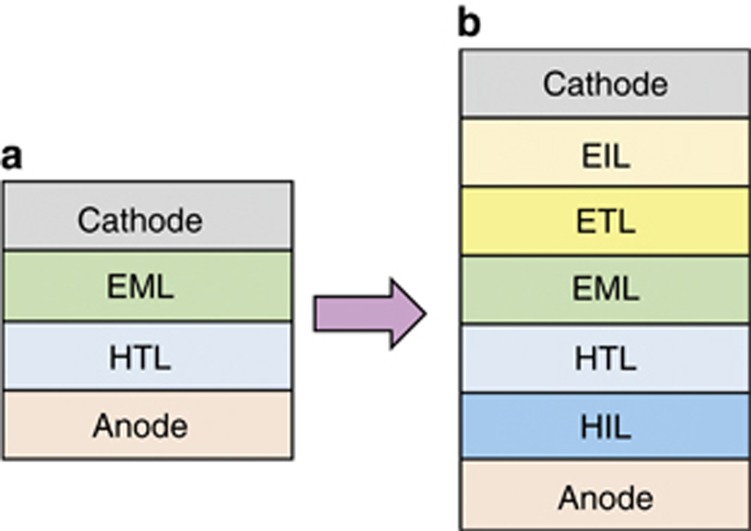
Schematic diagram of an OLED. (**a**) Basic structure proposed by Tang and VanSlyke in 1987. (**b**) Multi-layer structure employed in current OLED products. EIL, electron-injection layer; ETL, electron-transporting layer; EML, emitting layer; HTL, hole-transporting layer; HIL, hole-injection layer.

The basic structure of an OLED display, proposed by Tang and VanSlyke^41^ in 1987, consists of organic stacks sandwiched between anode and cathode, as shown in Figure 3a. Electrons and holes are injected from electrodes to organic layers for recombination and light emission; hence, an OLED display is an emissive display, unlike an LCD. Currently, multi-layer structures in OLEDs with different functional materials are commonly used, as shown in Figure 3b. The emitting layer (EML), which is used for light emission, consists of dopant and host materials with high quantum efficiency and high carrier mobility. Hole-transporting layer (HTL) and electron-transporting layer (ETL) between the EML and electrodes bring carriers into the EML for recombination. Hole- and electron-injection layers (HIL and EIL) are inserted between the electrodes and the HTL and ETL interface to facilitate carrier injection from the conductors to the organic layers. When applying voltage to the OLED, electrons and holes supplied from the cathode and anode, respectively, transport to the EML for recombination to give light.

Generally, each layer in an OLED is quite thin, and the total thickness of the whole device is <1 μm (substrates are not included). Thus the OLED is a perfect candidate for flexible displays. For an intrinsic organic material, its carrier mobility (<0.1 cm^2^ Vs^−1^) and free carrier concentration (10^10^ cm^−3^) are fairly low, limiting the device efficiency. Thus doping technology is commonly used^42, 43^. Additionally, to generate white light, two configurations can be considered: (1) patterned red, green and blue (RGB) OLEDs; and (2) a white OLED with RGB color filters (CFs). Both have pros and cons. In general, RGB OLEDs are mostly used for small-sized mobile displays, while white OLEDs with CFs are used for large TVs.

The EML is the core of an OLED. Based on the emitters inside, OLED devices can be categorized into four types: fluorescence, triplet-triplet fluorescence (TTF), phosphorescence, and thermally activated delayed fluorescence (TADF)^41, 44, 45, 46, 47^.

#### Fluorescent OLED

**Figure 4:**
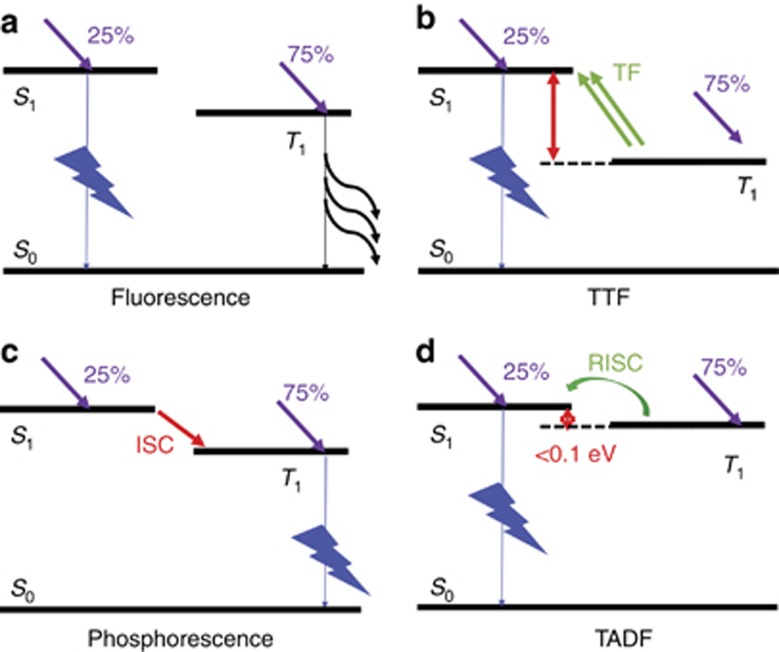
Illustration of the emission mechanisms of OLEDs: (**a**) fluorescence, (**b**) TTF, (**c**) phosphorescence, and (**d**) TADF. ISC, intersystem crossing; RISC, reverse intersystem crossing; TF, triplet fusion.

First, upon electrical excitation, 25% singlets and 75% triplets are formed with higher and lower energy, respectively. In a fluorescent OLED, only singlets decay radiatively through fluorescence with an ~ns exciton lifetime, which sets the theoretical limit of the internal quantum efficiency (IQE) to 25%, as shown in Figure 4a.

#### Triplet-triplet fluorescent OLED

Two triplet excitons may fuse to form one singlet exciton through the so-called triplet fusion process, as shown in Figure 4b, and relaxes to the energy from the singlet state, called TTF, which improves the theoretical limit of the IQE to 62.5%.

#### Phosphorescent OLED

With the introduction of heavy metal atoms (such as Ir and Pt) into the emitters, strong spin-orbital coupling greatly reduces the triplet lifetime to ~μs, which results in efficient phosphorescent emission. The singlet exciton experiences intersystem crossing to the triplet state for light emission, achieving a 100% IQE, as shown in Figure 4c. Owing to the long radiative lifetime (~μs) in a phosphorescent OLED, the triplet may interact with another triplet and polaron (triplet-triplet annihilation and triplet-polaron annihilation, respectively), which results in efficiency roll-off under high current driving^48^. Such processes may create hot excitons and hot polarons to shorten the operation lifetime, especially for blue-emitting devices, as will be discussed in the next section^49^.

#### Thermally activated delayed fluorescent OLED

The energy between the singlet and triplet can be reduced (<0.1 eV) by minimizing the exchange energy^50^; thus the triplet can jump back to the singlet state by means of thermal energy (reverse intersystem crossing) for fluorescence emission, which is called TADF, as shown in Figure 4d. Achieving a 100% IQE is possible for TADF emission without a heavy atom in the organic material, which reduces the material cost and is more flexible for organic molecular design.

In practical applications, red and green phosphorescent emitters are the mainstream for active matrix (AM) OLEDs due to their high IQE. While, for blue emitters, TTF is mostly used because of its longer operation lifetime^51^. However, recently, TADF materials have been rapidly emerging and are expected to have widespread applications in the near future.

It is worth mentioning that, although IQE could be as high as 100% in theory, due to the refractive index difference the emission generated inside the OLED experiences total internal reflection, which reduces the extraction efficiency. Taking a bottom emission OLED with a glass substrate (*n*~1.5) and an indium-tin-oxide anode (*n*~1.8) as an example, the final extraction efficiency is only ~20%^52^.

## Display metrics

To evaluate the performance of display devices, several metrics are commonly used, such as response time, CR, color gamut, panel flexibility, viewing angle, resolution density, peak brightness, lifetime, among others. Here we compare LCD and OLED devices based on these metrics one by one.

### Response time and motion picture response time

**Figure 5:**
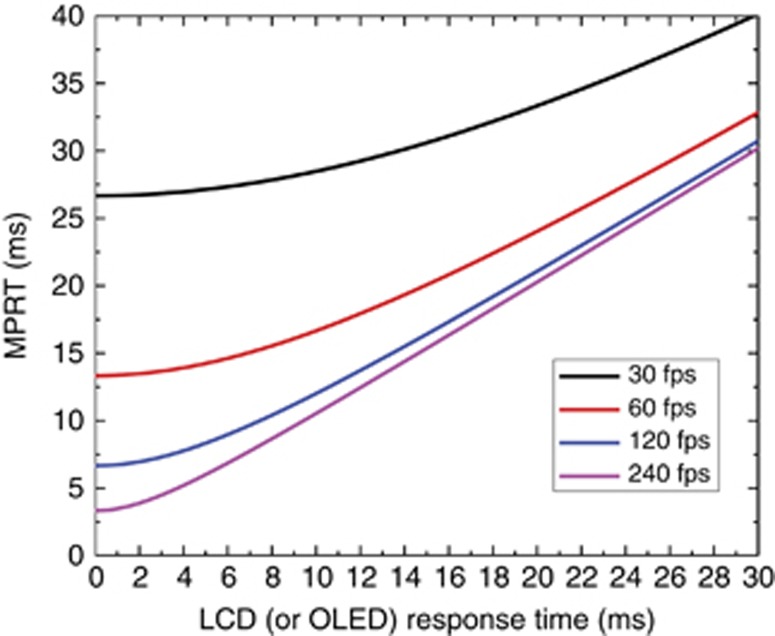
Calculated MPRT as a function of the LC (or OLED) response time at different frame rates.

A fast response time helps to mitigate motion image blur and boost the optical efficiency, but this statement is only qualitatively correct. When quantifying the visual performance of a moving object, motion picture response time (MPRT) is more representative, and the following equation should be used^53, 54, 55, 56, 57, 58^:


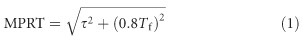


where *T*_f_ is the frame time (e.g., *T*_f_=16.67 ms for 60 fps). Using this equation, we can easily obtain an MPRT as long as the LC response time and TFT frame rate are known. The results are plotted in Figure 5.

From Figure 5, we can gain several important physical insights: (1) Increasing the frame rate is a simple approach to suppress image motion blur, but its improvement gradually saturates. For example, if the LC response time is 10 ms, then increasing the frame rate from 30 to 60 fps would significantly reduce the MPRT. However, as the TFT frame rate continues to increase to 120 and 240 fps, then the improvement gradually saturates. (2) At a given frame rate, say 120 fps, as the LC response time decreases, the MPRT decreases almost linearly and then saturates. This means that the MPRT is mainly determined by the TFT frame rate once the LC response time is fast enough, i.e., *τ*≪*T*_f_. Under such conditions, Equation (1) is reduced to MPRT≈0.8*T*_f_. (3) When the LC response is <2 ms, its MPRT is comparable to that of an OLED at the same frame rate, e.g., 120 fps. Here we assume the OLED’s response time is 0.

The last finding is somehow counter to the intuition that a LCD should have a more severe motion picture image blur, as its response time is approximately 1000 × slower than that of an OLED (ms vs. μs). To validate this prediction, Chen *et al.*^58^ performed an experiment using an ultra-low viscosity LC mixture in a commercial VA test cell. The measured average gray-to-gray response time is 1.29 ms by applying a commonly used overdrive and undershoot voltage method. The corresponding average MPRT at 120 fps is 6.88 ms, while that of an OLED is 6.66 ms. These two results are indeed comparable. If the frame rate is doubled to 240 fps, both LCDs and OLEDs show a much faster but still similar MPRT values (3.71 vs. 3.34 ms). Thus the above finding is confirmed experimentally.

If we want to further suppress image blur to an unnoticeable level (MPRT<2 ms), decreasing the duty ratio (for LCDs, this is the on-time ratio of the backlight, called scanning backlight or blinking backlight) is mostly adopted^59, 60, 61^. However, the tradeoff is reduced brightness. To compensate for the decreased brightness due to the lower duty ratio, we can boost the LED backlight brightness. For OLEDs, we can increase the driving current, but the penalties are a shortened lifetime and efficiency roll-off^62, 63, 64^.

### CR and ACR

High CR is a critical requirement for achieving supreme image quality. OLEDs are emissive, so, in theory, their CR could approach infinity to one. However, this is true only under dark ambient conditions. In most cases, ambient light is inevitable. Therefore, for practical applications, a more meaningful parameter, called the ACR, should be considered^65, 66, 67, 68^:


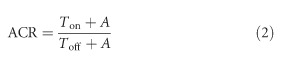


where *T*_on_ (*T*_off_) represents the on-state (off-state) brightness of an LCD or OLED and *A* is the intensity of reflected light by the display device.

**Figure 6:**
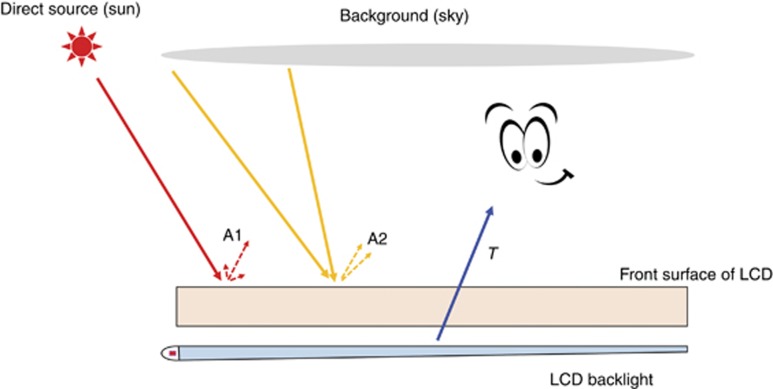
Schematic diagram of two types of reflections for an LCD (or OLED).

As Figure 6 depicts, there are two types of surface reflections. The first one is from a direct light source, i.e., the sun or a light bulb, denoted as A1. Its reflection is fairly specular, and in practice, we can avoid this reflection (i.e., strong glare from direct sun) by simply adjusting the display position or viewing direction. However, the second reflection, denoted as A2, is quite difficult to avoid. It comes from an extended background light source, such as a clear sky or scattered ceiling light. In our analysis, we mainly focus on the second reflection (A2).

**Figure 7:**
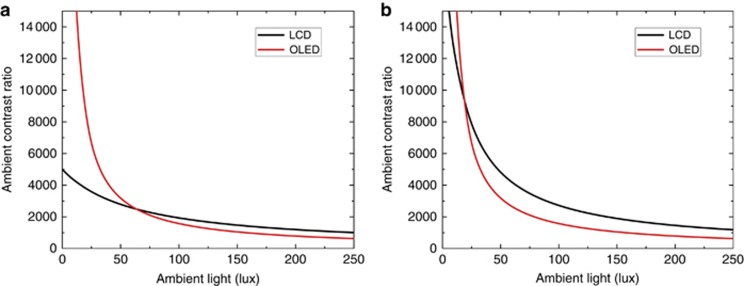
Calculated ACR as a function of different ambient light conditions for LCD and OLED TVs. Here we assume that the LCD peak brightness is 1200 nits and OLED peak brightness is 600 nits, with a surface reflectance of 1.2% for both the LCD and OLED. (**a**) LCD CR: 5000:1, OLED CR: infinity; (**b**) LCD CR: 20 000:1, OLED CR: infinity.

To investigate the ACR, we have to clarify the reflectance first. A large TV is often operated by remote control, so touchscreen functionality is not required. As a result, an anti-reflection coating is commonly adopted. Let us assume that the reflectance is 1.2% for both LCD and OLED TVs. For the peak brightness and CR, different TV makers have their own specifications. Here, without losing generality, let us use the following brands as examples for comparison: LCD peak brightness=1200 nits, LCD CR=5000:1 (Sony 75″ X940E LCD TV); OLED peak brightness=600 nits, and OLED CR=infinity (Sony 77″ A1E OLED TV). The obtained ACR for both LCD and OLED TVs is plotted in Figure 7a. As expected, OLEDs have a much higher ACR in the low illuminance region (dark room) but drop sharply as ambient light gets brighter. At 63 lux, OLEDs have the same ACR as LCDs. Beyond 63 lux, LCDs take over. In many countries, 60 lux is the typical lighting condition in a family living room. This implies that LCDs have a higher ACR when the ambient light is brighter than 60 lux, such as in office lighting (320–500 lux) and a living room with the window shades or curtain open. Please note that, in our simulation, we used the real peak brightness of LCDs (1200 nits) and OLEDs (600 nits). In most cases, the displayed contents could vary from black to white. If we consider a typical 50% average picture level (i.e., 600 nits for LCDs vs. 300 nits for OLEDs), then the crossover point drops to 31 lux (not shown here), and LCDs are even more favorable. This is because the on-state brightness plays an important role to the ACR, as Equation (2) shows.

Recently, an LCD panel with an in-cell polarizer was proposed to decouple the depolarization effect of the LC layer and color filters^69^. Thus the light leakage was able to be suppressed substantially, leading to a significantly enhanced CR. It has been reported that the CR of a VA LCD could be boosted to 20 000:1. Then we recalculated the ACR, and the results are shown in Figure 7b. Now, the crossover point takes place at 16 lux, which continues to favor LCDs.

**Figure 8:**
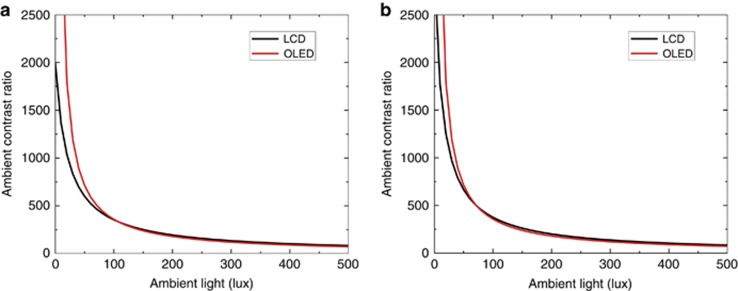
Calculated ACR as a function of different ambient light conditions for LCD and OLED smartphones. Reflectance is assumed to be 4.4% for both LCD and OLED. (**a**) LCD CR: 2000:1, OLED CR: infinity; (**b**) LCD CR: 3000:1, OLED CR: infinity. (LCD peak brightness: 600 nits; OLED peak brightness: 500 nits).

For mobile displays, such as smartphones, touch functionality is required. Thus the outer surface is often subject to fingerprints, grease and other contaminants. Therefore, only a simple grade AR coating is used, and the total surface reflectance amounts to ~4.4%. Let us use the FFS LCD as an example for comparison with an OLED. The following parameters are used in our simulations: the LCD peak brightness is 600 nits and CR is 2000:1, while the OLED peak brightness is 500 nits and CR is infinity. Figure 8a depicts the calculated results, where the intersection occurs at 107 lux, which corresponds to a very dark overcast day. If the newly proposed structure with an in-cell polarizer is used, the FFS LCD could attain a 3000:1 CR^69^. In that case, the intersection is decreased to 72 lux (Figure 8b), corresponding to an office building hallway or restroom lighting. For reference, a typical office light is in the range of 320–500 lux^70^. As Figure 8 depicts, OLEDs have a superior ACR under dark ambient conditions, but this advantage gradually diminishes as the ambient light increases. This was indeed experimentally confirmed by LG Display^71^. Display brightness and surface reflection have key roles in the sunlight readability of a display device.

### Color gamut

Vivid color is another critical requirement of all display devices^72^. Until now, several color standards have been proposed to evaluate color performance, including sRGB, NTSC, DCI-P3 and Rec. 2020^73, 74, 75, 76^. It is believed that Rec. 2020 is the ultimate goal, and its coverage area in color space is the largest, nearly twice as wide as that of sRGB. However, at the present time, only RGB lasers can achieve this goal.

**Figure 9:**
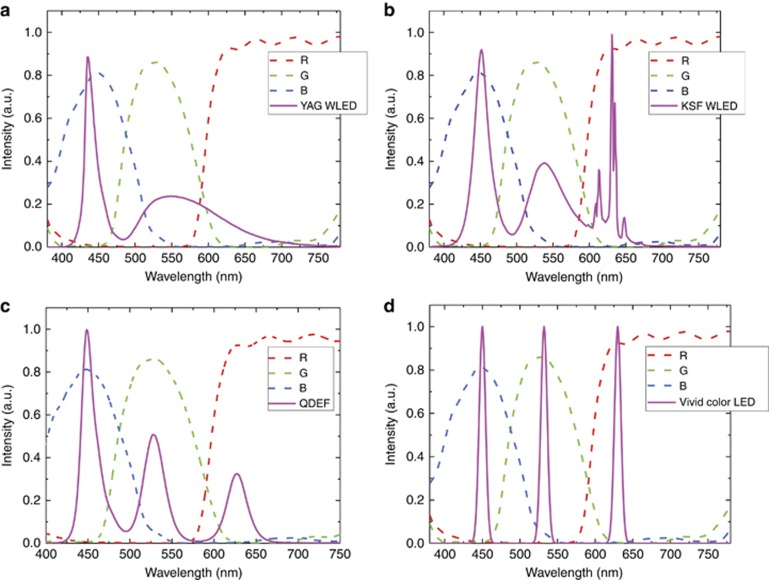
Transmission spectra of color filters and emission spectra of (**a**) YAG WLED, (**b**) KSF WLED, (**c**) QDEF and (**d**) Vivid Color LED. KSF, potassium silicofluoride; QDEF, quantum dot enhancement film; WLED, white light-emitting diode; YAG, yttrium aluminum garnet.

For conventional LCDs employing a WLED backlight, the yellow spectrum generated by YAG (yttrium aluminum garnet) phosphor is too broad to become highly saturated RGB primary colors, as shown in Figure 9a^77^. As a result, the color gamut is only ~50% Rec. 2020. To improve the color gamut, more advanced backlight units have been developed, as summarized in Table 2. The first choice is the RG-phosphor-converted WLED^78, 79^. From Figure 9b, the red and green emission spectra are well separated; still, the green spectrum (generated by β-sialon:Eu^2+^ phosphor) is fairly broad and red spectrum (generated by K_2_SiF_6_:Mn^4+^ (potassium silicofluoride, KSF) phosphor) is not deep enough, leading to 70%–80% Rec. 2020, depending on the color filters used.

A QD-enhanced backlight (e.g., quantum dot enhancement film, QDEF) offers another option for a wide color gamut^20, 80, 81^. QDs exhibit a much narrower bandwidth (FWHM~20–30 nm) (Figure 9c), so that high purity RGB colors can be realized and a color gamut of ~90% Rec. 2020 can be achieved. One safety concern is that some high-performance QDs contain the heavy metal Cd. To be compatible with the restriction of hazardous substances, the maximum cadmium content should be under 100 ppm in any consumer electronic product^82^. Some heavy-metal-free QDs, such as InP, have been developed and used in commercial products^83, 84, 85^.

Recently, a new LED technology, called the Vivid Color LED, was demonstrated^86^. Its FWHM is only 10 nm (Figure 9d), which leads to an unprecedented color gamut (~98% Rec. 2020) together with specially designed color filters. Such a color gamut is comparable to that of laser-lit displays but without laser speckles. Moreover, the Vivid Color LED is heavy-metal free and shows good thermal stability. If the efficiency and cost can be further improved, it would be a perfect candidate for an LCD backlight.

The color performance of a RGB OLED is mainly governed by the three independent RGB EMLs. Currently, both deep blue fluorescent OLEDs^87^ and deep red phosphorescent OLEDs^88^ have been developed. The corresponding color gamut is >90% Rec. 2020. Apart from material development^89^, the color gamut of OLEDs could also be enhanced by device optimization. For example, a strong cavity could be formed between a semitransparent and reflective layer. This selects certain emission wavelengths and hence reduces the FWHM^90^. However, the tradeoff is increased color shift at large viewing angles^91^.

A color filter array is another effective approach to enhance the color gamut of an OLED. For example, in 2017, AUO demonstrated a 5-inch top-emission OLED panel with 95% Rec. 2020. In this design, so-called symmetric panel stacking with a color filter is employed to generate purer RGB primary colors^92^. Similarly, SEL developed a tandem white top-emitting OLED with color filters to achieve a high color gamut (96% Rec. 2020) and high resolution density (664 pixels per inch (ppi) simultaneously^93^.

### Lifetime

As mentioned earlier, TFT LCDs are a fairly mature technology. They can be operated for >10 years without noticeable performance degradation. However, OLEDs are more sensitive to moisture and oxygen than LCDs. Thus their lifetime, especially for blue OLEDs, is still an issue. For mobile displays, this is not a critical issue because the expected usage of a smartphone is approximately 2–3 years. However, for large TVs, a lifetime of >30 000 h (>10 years) has become the normal expectation for consumers.

Here we focus on two types of lifetime: storage and operational. To enable a 10-year storage lifetime, according to the analysis^94^, the water vapor permeation rate and oxygen transmission rate for an OLED display should be <1 × 10^−6^ g (m^2^-day)^−1^ and 1 × 10^−5^ cm^3^ (m^2^-day)^−1^, respectively. To achieve these values, organic and/or inorganic thin films have been developed to effectively protect the OLED and lengthen its storage lifetime. Meanwhile, it is compatible to flexible substrates and favors a thinner display profile^95, 96, 97^.

The next type of lifetime is operational lifetime. Owing to material degradation, OLED luminance will decrease and voltage will increase after long-term driving^98^. For red, yellow and green phosphorescent OLEDs, their lifetime values at 50% luminance decrease (*T*_50_) can be as long as >80 000 h with a 1000 cd m^−2^ luminance^99, 100, 101^. Nevertheless, the operational lifetime of the blue phosphor is far from satisfactory. Owing to the long exciton lifetime (~μs) and wide bandgap (~3 eV), triplet-polaron annihilation occurs in the blue phosphorescent OLED, which generates hot polarons (~6 eV; this energy is higher than some bond energies, e.g., 3.04 eV for the C-N single bond), leading to a short lifetime. To improve its lifetime, several approaches have been proposed, such as designing a suitable device structure to broaden the recombination zone, stacking two or three OLEDs or introducing an exciton quenching layer. The operation lifetime of a blue phosphorescent OLED can be improved to 3700 h (*T*_50_, half lifetime) with an initial luminance of 1000 nits. However, this is still ~20 × shorter than that of red and green phosphorescent OLEDs^101, 102, 103^.

**Figure 10:**
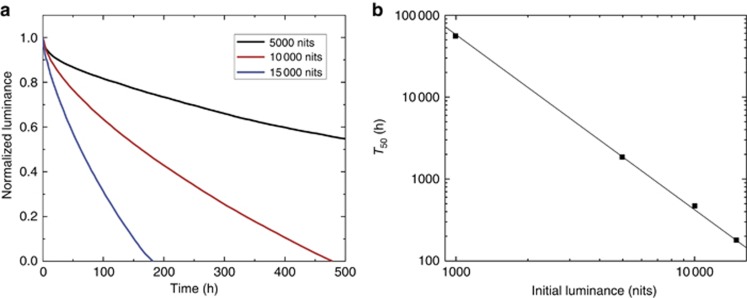
(**a**) Luminance decay curves for the blue OLED with different initial luminance values. (**b**) Estimated *T*_50_ under different initial luminance values.

To further enhance the lifetime of the blue OLED, the NTU group has developed new ETL and TTF-EML materials together with an optimized layer structure and double EML structure^104^. Figure 10a shows the luminance decay curves of such a blue OLED under different initial luminance values (5000, 10 000, and 15 000 nits). From Figure 10b, the estimated *T*_50_ at 1000 nits of this blue OLED is ~56 000 h (~6–7 years)^104, 105^. As new materials and novel device structures continue to advance, the lifetime of OLEDs will be gradually improved.

### Power efficiency

Power consumption is equally important as other metrics. For LCDs, power consumption consists of two parts: the backlight and driving electronics. The ratio between these two depends on the display size and resolution density. For a 55″ 4K LCD TV, the backlight occupies approximately 90% of the total power consumption. To make full use of the backlight, a dual brightness enhancement film is commonly embedded to recycle mismatched polarized light^106^. The total efficiency could be improved by ~60%.

**Figure 11:**
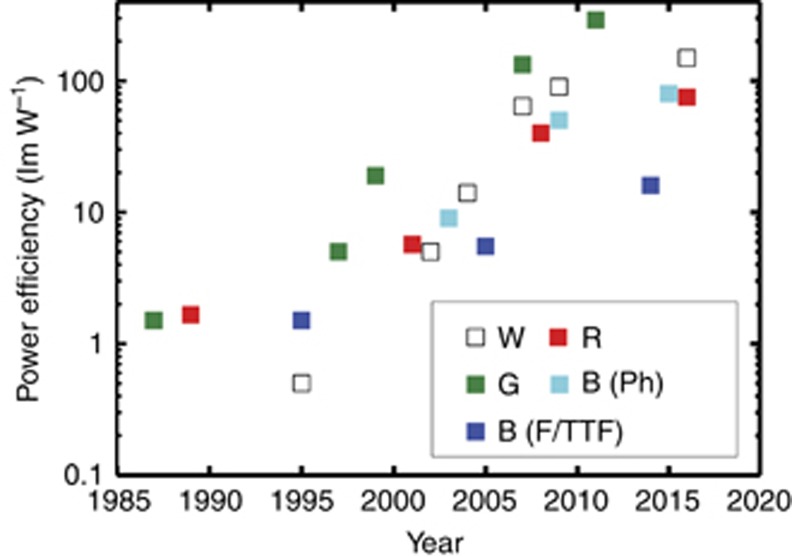
Power efficiency of white, red, green and phosphorescent blue and fluorescent/TTF blue OLEDs over time. Data are compiled from Refs. 41, 45, 114, 115, 116, 117, 118, 119, 120, 121, 122, 123, 124, 125, 126, 127, 128, 129, 130, 131, 132, 133.

The power efficiency of an OLED is generally limited by the extraction efficiency (*η*_ext_~20%). To improve the power efficiency, multiple approaches can be used, such as a microlens array, a corrugated structure with a high refractive index substrate^107^, replacing the metal electrode (such as the Al cathode) with a transparent metal oxide^108^, increasing the distance from the emission dipole to the metal electrode^109^ or increasing the carrier concentration by material optimizations^110^. Experimentally, external quantum efficiencies as high as 63% have been demonstrated^107, 108^. Note that sometimes the light-extraction techniques result in haze and image blur, which deteriorate the ACR and display sharpness^111, 112, 113^. Additionally, fabrication complexity and production yield are two additional concerns. Figure 11 shows the power efficiencies of white, green, red and blue phosphorescent as well as blue fluorescent/TTF OLEDs over time. For OLEDs with fluorescent emitters in the 1980s and 1990s, the power efficiency was limited by the IQE, typically <10 lm W^−1^(Refs. 41, 114, 115, 116, 117, 118). With the incorporation of phosphorescent emitters in the ~2000 s, the power efficiency was significantly improved owing to the materials and device engineering^45, 119, 120, 121, 122, 123, 124, 125^. The photonic design of OLEDs with regard to the light extraction efficiency was taken into consideration for further enchantment of the power efficiency^126, 127, 128, 129, 130^. For a green OLED, a power efficiency of 290 lm W^−1^ was demonstrated in 2011 (Ref. 127), which showed a >100 × improvement compared with that of the basic two-layer device proposed in 1987 (1.5 lm W^−1^ in Ref. 41). A white OLED with a power efficiency >100 lm W^−1^ was also demonstrated, which was comparable to the power efficiency of a LCD backlight. For red and blue OLEDs, their power efficiencies are generally lower than that of the green OLED due to their lower photopic sensitivity function, and there is a tradeoff between color saturation and power efficiency. Note, we separated the performances of blue phosphorescent and fluorescent/TTF OLEDs. For the blue phosphorescent OLEDs, although the power efficiency can be as high as ~80 lm W^−1^, the operation lifetime is short and color is sky-blue. For display applications, the blue TTF OLED is the favored choice, with an acceptable lifetime and color but a much lower power efficiency (16 lm W^−1^) than its phosphorescent counterpart^131, 132^. Overall, over the past three decades (1987–2017), the power efficiency of OLEDs has improved dramatically, as Figure 11 shows.

To compare the power consumption of LCDs and OLEDs with the same resolution density, the displayed contents should be considered as well. In general, OLEDs are more efficient than LCDs for displaying dark images because black pixels consume little power for an emissive display, while LCDs are more efficient than OLEDs at displaying bright images. Currently, a ~65% average picture level is the intersection point between RGB OLEDs and LCDs^134^. For color-filter-based white OLED TVs, this intersection point drops to ~30%. As both technologies continue to advance, the crossover point will undoubtedly change with time.

### Panel flexibility

Flexible displays have a long history and have been attempted by many companies, but this technology has only recently begun to see commercial implementations for consumer electronics^135^. A good example is Samsung’s flagship smartphone, the Galaxy S series, which has an OLED display panel that covers the edge of the phone. However, strictly speaking, it is a curved display rather than a flexible display. One step forward, a foldable AM-OLED has been demonstrated with the curvature radius of 2 mm for 100 000 repeated folds^136^. Owing to the superior flexibility of the organic materials, a rollable AM-OLED display driven by an organic TFT was fabricated^137^. By replacing the brittle indium-tin-oxide with a flexible Ag nanowire as the anode, a stretchable OLED for up to a 120% strain was demonstrated^138^.

LCDs have limited flexibility. A curved TV is practical but going beyond that is rather difficult with rigid and thick glass substrates^139^. Fortunately, this obstacle has been removed with the implementation of a thin plastic substrate^140, 141, 142^. In 2017, a 12.1″ rollable LCD using organic TFT, called OLCD, was demonstrated, and its radius of curvature is 60 mm^143^. To maintain a uniform cell gap, a polymer wall was formed within the LC layer^144^. Additionally, it is reported that LCDs could be foldable with a segmented backlight. This is a good choice, but until now, no demo or real device has been demonstrated. Combining two bezel-less LCDs together is another solution to enable a foldable display, but this technology is still under development^145^.

### Others

In addition to the aforementioned six display metrics, other parameters are equally important. For example, high-resolution density has become a standard for all high-end display devices. Currently, LCD is taking the lead in consumer electronic products. Eight-hundred ppi or even >1000 ppi LCDs have already been demonstrated and commercialized, such as in the Sony 5.5″ 4k Smartphone Xperia Z5 Premium. The resolution of RGB OLEDs is limited by the physical dimension of the fine-pitch shadow mask. To compete with LCDs, most OLED displays use the PenTile RGB subpixel matrix scheme^146^. The effective resolution density of an RGB OLED mobile display is~500 ppi. In the PenTile configuration, the blue subpixel has a larger size than the green and red subpixels. Thus a lower current is needed to achieve the required brightness, which is helpful for improving the lifetime of the blue OLED. On the other hand, owing to the lower efficiency of the blue TTF OLED compared with the red and green phosphorescent ones, this results in higher power consumption. To further enhance the resolution density, multiple approaches have been developed, as will be discussed later.

The viewing angle is another important property that defines the viewing experience at large oblique angles, which is quite critical for multi-viewer applications. OLEDs are self-emissive and have an angular distribution that is much broader than that of LCDs. For instance, at a 30° viewing angle, the OLED brightness only decreases by 30%, whereas the LCD brightness decrease exceeds 50%. To widen an LCD’s viewing angle, three options can be used. (1) Remove the brightness-enhancement film in the backlight system. The tradeoff is decreased on-axis brightness^147^. (2) Use a directional backlight with a front diffuser^148, 149^. Such a configuration enables excellent image quality regardless of viewing angle; however, image blur induced by a strong diffuser should be carefully treated. (3) Use QD arrays as the color filters^20, 150, 151, 152^. This design produces an isotropic viewing cone and high-purity RGB colors. However, preventing ambient light excitation of QDs remains a technical challenge^20^.

In addition to brightness, color, grayscale and the CR also vary with the viewing angle, known as color shift and gamma shift. In these aspects, LCDs and OLEDs have different mechanisms. For LCDs, they are induced by the anisotropic property of the LC material, which could be compensated for with uniaxial or biaxial films^5^. For OLEDs, they are caused by the cavity effect and color-mixing effect^153, 154^. With extensive efforts and development, both technologies have fairly mature solutions; currently, color shift and gamma shift have been minimized at large oblique angles.

Cost is another key factor for consumers. LCDs have been the topic of extensive investigation and investment, whereas OLED technology is emerging and its fabrication yield and capability are still far behind LCDs. As a result, the price of OLEDs is about twice as high as that of LCDs, especially for large displays. As more investment is made in OLEDs and more advanced fabrication technology is developed, such as ink-jet printing^155, 156, 157^, their price should decrease noticeably in the near future.

## Future perspectives

Currently, both LCDs and OLEDs are commercialized and compete with each other in almost every display segment. They are basically two different technologies (non-emissive vs. emissive), but as a display, they share quite similar perspectives in the near future. Here we will focus on three aspects: HDR, VR/AR and smart displays with versatile functions.

### High dynamic range

HDR is an emerging technology that can significantly improve picture quality^158, 159, 160^. However, strictly speaking, HDR is not a single metric; instead, it is more like a technical standard or a format (e.g., HDR10, Dolby Vision, etc.), unifying the aforementioned metrics. In general, HDR requires a higher CR (CR≥100 000:1), deeper dark state, higher peak brightness, richer grayscale (≥10 bits) and more vivid color.

Both LCD and OLED are HDR-compatible. Currently, the best HDR LCDs can produce brighter highlights than OLEDs, but OLEDs have better overall CRs thanks to their superior black level. To enhance an LCD’s CR, a local dimming backlight is commonly used, but its dimming accuracy is limited by the number of LED segmentations^161, 162, 163^. Recently, a dual-panel LCD system was proposed for pixel-by-pixel local dimming^164, 165^. In an experiment, an exceedingly high CR (>1 000 000:1) and high bit-depth (>14 bits) were realized at merely 5 volts. In 2017, such a dual-panel LCD was demonstrated by Panasonic, aiming at medical and vehicular applications. At 2018 consumer electronics show, Innolux demonstrated a 10.1″ LCD with an active matrix mini-LED backlight. The size of each mini-LED is 1 mm and pitch length is 2 mm. In total, there are 6720 local dimming zones. Such a mini-LED based LCD offers several attractive features: CR>1 000 000:1, peak brightness=1500 nits, HDR: 10-bit mini-LED and 8-bit LCD, and thin profile.

Also worth mentioning here is ultra-high brightness. Mostly, people pay more attention to the required high CR (CR>100 000:1) of HDR but fail to notice that CR is jointly determined by the dark state and peak brightness. For example, a 12-bit Perceptual Quantizer curve is generated for a range up to 10 000 nits, which is far beyond what current displays can provide^166, 167^.

The peak brightness of LCDs could be boosted to 2000 nits or even higher by simply using a high-power backlight. OLEDs are self-emissive, so their peak brightness would trade with lifetime. As a result, more advanced OLED materials and novel structural designs are highly desirable in the future. Another reason to boost peak brightness is to increase sunlight readability. Especially for some outdoor applications, such as public displays, peak brightness is critical to ensure good readability under strong ambient light. As discussed in the section of ‘CR and ACR’, high brightness leads to a high ACR, except that the power consumption will increase.

### Virtual reality and augmented reality

Immersive VR/AR are two emerging wearable display technologies with great potential in entertainment, education, training, design, advertisement and medical diagnostics. However, new opportunities arise along with new challenges. VR head-mounted displays require a resolution density as high as >2000 ppi to eliminate the so-called screen door effect and generate more realistic immersive experiences.

An LCD’s resolution density is determined by the TFTs and color filter arrays. In SID 2017, Samsung demonstrated an LCD panel with a resolution of 2250 ppi for VR applications. The pitches of the sub-pixel and pixel are 3.76 and 11.28 μm, respectively. Meanwhile, field sequential color provides another promising option to triple the LCD resolution density^168, 169^. However, more advanced LC mixtures and fast response LCD modes are needed to suppress the color breakup issue^170, 171, 172, 173, 174, 175, 176, 177, 178, 179^. For OLED microdisplays, eMagin proposed a novel direct patterning approach to enable 2645 ppi RGB organic emitters on a CMOS backplane^180^. Similar performance has been obtained by Sony. They developed a 0.5-inch AM-OLED panel with 3200 ppi using well-controlled color filter arrays^181^.

As for AR applications, lightweight, low power and high brightness are mainly determined by the display components. LC on silicon can generate high brightness^182^, but its profile is too bulky and heavy with the implementation of a polarization beam splitter. Removing the polarization beam splitter with a front light guide would be the appropriate solution^183^. However, integrating RGB LEDs with this light guide remains a significant challenge. Additionally, RGB LEDs, especially green LEDs, are not efficient enough. OLEDs have thin profiles, but their peak brightness and power efficiency are still far from satisfactory, especially for such AR devices, as they are mostly used outdoors, meaning high brightness is commonly required to increase the ACR of displayed images.

### Smart displays with versatile functions

Currently, displays are no longer limited to traditional usages, such as TVs, pads or smartphones. Instead, they have become more diversified and are used in smart windows, smart mirrors, smart fridges, smart vending machines and so on. They have entered all aspects of our daily lives.

As these new applications are emerging, LCDs and OLEDs have new opportunities as well as new challenges. Let us take a vehicle display as an example: high brightness, good sunlight readability, and a wide working temperature range are required^184^. To follow this trend, LC mixtures with an ultra-high clearing temperature (>140 °C) have been recently developed, ensuring that the LCD works properly even at some extreme temperatures^185^. OLEDs have an attractive form factor for vehicle displays, but their performance needs to qualify under the abovementioned harsh working conditions. Similarly, for transparent displays or mirror displays, LCDs and OLEDs have their own merits and demerits^186, 187, 188, 189^. They should aim at versatile functions based on their own strengths.

## Conclusion

We have briefly reviewed the recent progress of LCD and OLED technologies. Each technology has its own pros and cons. For example, LCDs are leading in lifetime, cost, resolution density and peak brightness; are comparable to OLEDs in ACR, viewing angle, power consumption and color gamut (with QD-based backlights); and are inferior to OLED in black state, panel flexibility and response time. Two concepts are elucidated in detail: the motion picture response time and ACR. It has been demonstrated that LCDs can achieve comparable image motion blur to OLEDs, although their response time is 1000 × slower than that of OLEDs (ms vs. μs). In terms of the ACR, our study shows that LCDs have a comparable or even better ACR than OLEDs if the ambient illuminance is >50 lux, even if its static CR is only 5000:1. The main reason is the higher brightness of LCDs. New trends for LCDs and OLEDs are also highlighted, including ultra-high peak brightness for HDR, ultra-high-resolution density for VR, ultra-low power consumption for AR and ultra-versatile functionality for vehicle display, transparent display and mirror display applications. The competition between LCDs and OLEDs is still ongoing. We believe these two TFT-based display technologies will coexist for a long time.

## Figures and Tables

**Table 1:** Performance comparisons of four popular LCD modes

	TN mode	MVA mode	IPS mode	FFS mode
Transmittance (normalized to TN)	100%	70%–80%	70%–80%	88%–98%
On-axis contrast ratio	~1000:1	~5000:1	~2000:1	~2000:1
LC mixture	+Δ*ε*	−Δ*ε*	+Δ*ε* or −Δ*ε*	+Δ*ε* or −Δ*ε*
Viewing angle	Fair	Good	Excellent	Excellent
Response time	~5 ms	~5 ms	~10 ms	~10 ms
Touch panel	No	No	Yes	Yes
Applications	Wristwatches, signage, laptop computers	TV, desktop computers	Desktop computers, pads	Smartphones, pads, notebook computers

Abbreviations: FFS, fringe-field switching; IPS, in-plane switching; LCD, liquid crystal display; MVA, multi-domain vertical alignment; TN, twisted nematic; TV, television.

**Table 2:** Comparison of different light sources in LCD backlights

	YAG WLED	KSF WLED	QDEF a	Vivid Color LED
FWHM	>100 nm	55 nm for green 2 nm for red (5 peaks)	20–30 nm	10 nm
Tunability	No	No	Yes	Yes
Color gamut	~50% Rec. 2020	70%–80% Rec. 2020	80%–90% Rec. 2020	>90% Rec. 2020
Efficiency	High	High	Moderate	Low
Cost	Low	Moderate	High	High
Stability	Excellent	Good	Good	Excellent
RoHS	Yes	Yes	Cd-based	Yes

Abbreviations: FWHM, full width at half maximum; KSF, potassium silicofluoride; LED, light-emitting diode; QDEF, quantum dot enhancement film; RoHS, restriction of hazardous substances; WLED, white light-emitting diode; YAG, yttrium aluminum garnet.

a Here we only consider Cd-based quantum-dots (QDs). For heavy-metal-free QDs, e.g., InP QD, the FWHM is broader (40–50 nm) and color gamut is 70–80%. Their optical efficiency is slightly lower than that of Cd-based QDs.
